# Xiaoyao Kangai Jieyu Fang, a Chinese Herbal Formulation, Ameliorates Cancer-Related Depression Concurrent with Breast Cancer in Mice via Promoting Hippocampal Synaptic Plasticity

**DOI:** 10.1155/2018/3967642

**Published:** 2018-11-18

**Authors:** Pan Meng, Yuanshan Han, Qin Yang, Hui Yang, Qing Zhu, Xiaoyuan Lin, Xiuli Zhang, Hongqing Zhao, Zhuo Liu, Jian Liu, Lin Tang, Weixu Luo, Yuhong Wang

**Affiliations:** ^1^Institute of Innovation and Applied Research in Chinese Medicine, Hunan University of Chinese Medicine, Changsha, Hunan 410208, China; ^2^The First Affiliated Hospital, Hunan University of Chinese Medicine, Changsha, Hunan 410007, China; ^3^The Affiliated Cancer Hospital of Xiangya School of Medicine, Central South University, Changsha, Hunan 410006, China

## Abstract

Diagnosis with breast cancer is a major life event that elicits increases in depressive symptoms for up to 50% of women. Xiaoyao Kangai Jieyu Fang (XYKAJY) is derived from a canonical TCM formula, Xiaoyao San (XYS), which has a history of nearly 1000 years for treating depression. The aim of this study was to investigate whether XYKAJY alleviates depression-like behavior and breast tumor proliferation in breast cancer mice then explore the mechanisms underlying its action on HPA axis and hippocampal plasticity further. XYKAJY was treated at the high dose of 1.95 g/mL and 0.488 g/mL, after 21 days of administration. Different behaviors, monoamine neurotransmitters, tumor markers, and the index of HPA axis were detected to evaluate depressive-like symptoms of breast cancer mice. Also, the pathological changes of the tumor, hippocampus, and the expressions of GR, NR2A, NR2B, CAMKII, CREB, and BDNF were detected. In this study, XYKAJY formulation significantly improved the autonomic behavior, reduced the incubation period of feeding, and reversed the typical depressive-like symptoms in breast cancer mice. Also, it reduced the content of CORT, ACTH, CRH, and CA125, CA153, CEA in the blood, protected the pathological changes of the hippocampus and tumor, upregulated the expression of GR, CREB, and BDNF in the hippocampus, and significantly decreased the expression of NR2A, NR2B, and CaMKII. These results provide direct evidence that XYKAJY effectively alleviates depression-like behaviors and tumor proliferation in vehicle mice with ameliorates hippocampus synaptic plasticity dysfunctions.

## 1. Introduction

Breast cancer is a common type of cancers among women. Many women experience clinically significant depression following the diagnosis of breast cancer. Women patients with breast cancer are 25-30% more likely to be diagnosed with a mood disorder (e.g., depression, anxiety) compared to the general population [1]. Moreover, 58% of cancer patients suffer from depression [2]. We should pay attention to cancer-related depression concurrent with breast cancer, as this comorbidity reduces quality of life, decreases treatment compliance, and promotes cancer progression [3, 4]. Therefore, investigation of the cancer-related depression diagnosis and treatment becomes very important.

The morbidity of cancer-related depression concurrent with breast cancer is related to the self-psychological adjustment, and also it is influenced by society, family, age, economy, education, the number of radiotherapy and so on. At present, its mechanism has not been clarified. Hypothalamus–pituitary–adrenocortical (HPA) axis is the reflection of the stress level in the body. HPA axis functioning predicted depressive symptoms in the context of breast cancer, and elevated cortisol in the evening was associated with depressive symptoms among breast cancer survivors over 14 months [5]. Elevated cortisol awakening response may be a key factor for increasing in depressive symptoms after breast cancer treatment [6]. CORT enters the brain through the blood-brain barrier and further affects the morphology and function of the hippocampus [7], which directly leads to synaptic plasticity injury even leading to the occurrence of depression [8].

Combination of chemotherapeutics and antidepressants is the currently prevalent therapeutic strategy for cancer-related depression concurrent with breast cancer [9]. Paclitaxel and tamoxifen are the most commonly used chemotherapeutic drugs for treating breast cancer. Specifically, paclitaxel causes chemotherapy-induced peripheral neuropathy, and nerve fiber dysfunction and degeneration exist, which could induce depression [10]. Selective serotonin reuptake inhibitors (SSRIs) are a group of antidepressant medications, which are widely used in moderate/severe depression [11]. Despite their widespread use, there have long been concerns that SSRIs may promote breast cancer by increasing prolactin levels [12, 13], which is an accepted risk factor for tumor progression [14, 15]. More recently, SSRIs have been shown to increase the rate of brain metastases in breast cancer mouse models, by altering the permeability of the blood-brain barrier [16]. Therefore, combined with chemotherapeutic and antidepressant drugs belonging to passive treatment, many shortcomings also exist [17]. Regarding its effects on various receptors, there are adverse reactions such as arrhythmia, hypertension, anticholinergic action, with the body discomfort caused by cancer itself, drug combination induce greatly reduces the compliance of the patients, further increase the psychological burden of patients and difficulty of treatment.

Traditional Chinese medicine (TCM) can improve the overall coordination about physical symptoms and mental disorders through multitargets, which is based on the whole dialectical theory of governance, with less toxicity and side effects. Many effective anticancer drugs have been discovered in clinic. Xiaoyao Kangai Jieyu Fang (XYKAJY) is derived from a canonical TCM formula, Xiaoyao San (XYS) which has a history of nearly 1000 years from “Taiping benevolent dispensary.” XYS is a valuable traditional Chinese prescription of antidepression in classical medicine with the functions of soothing liver and strengthening spleen and nourishing blood for regulating menstruation [18, 19]. XYKAJY is added and subtracted on the basis of XYS. In the current study, we investigated effects of XYKAJY on cancer-related depression concurrent with breast cancer and the underlying mechanisms of action of XYKAJY by focusing on HPA axis and hippocampal synaptic plasticity.

## 2. Materials and Methods

### 2.1. Animals

Sixty female 8- to 9-week old Balb/c mice were housed in 5/cage and acclimated to the temperature-controlled (22 ± 1°C) and humidity-controlled (55%-65% relative humidity) for 1 week under a 14:10 light:dark cycle (lights off at 15:00). The animals had free access to filtered water and rodent chow throughout the study and cotton nestlets and plastic huts were provided for nesting. All animal experiments were performed in accordance with the guidelines for the Care and Use of Laboratory Animals, and experimental protocols were approved by the committee of Ethics of Animal Experimentation of Hunan University of Chinese Medicine. All efforts were made to minimize animal suffering and to reduce the number of mice used.

### 2.2. Drug and Reagent

Raw materials of XYKAJY were purchased and concentrated to oral liquid (0.975 g/mL) in the First Affiliated Hospital of Hunan University of Chinese Medicine. The consists of XYKAJY formulation are shown in Table 1. Fluoxetine hydrochloride capsules (20 mg) were purchased from Hunan Xiangya Pharmaceutical and Patheon, France. Corticosterone was purchased from Sigma-Aldrich Co., Ltd. (St Louis, MO, USA).

### 2.3. Surgery, and Drug Administration

4T1 cells were obtained from Procell Life Science & Technology Co., Ltd. in Wuhan, China. These cell lines are separated from a single spontaneous Balb/c fC3H mammary tumor. The concentration of 10^7^ /ml 4T1 inflammatory cells was inoculated with 0.1mL in the armpit of Balb/c mice. After 7 days of inoculation, when the tumor was observed obviously, then we injected the 30 mg/kg corticosterone suspension (5 corticosterone 1/g was dissolved by 5 mL DMSO in the hypodermic, and ultrasound was used to dissolve the corticosterone in DMSO) for 21 days. The method was used to establish the vehicle mice of cancer-related depression concurrent with breast cancer.

The mice were adapted for 5 days, then randomly divided into five groups: (1) control and vehicle group: all mice were treated with distilled water; (2) paclitaxel combined fluoxetine group: the group was injected with liposomal paclitaxel with 20 ml/kg once a week, which was made of 1mg/ml mixture by 5% glucose, also with intragastric administration fluoxetine suspension for 21 days at the same time (the concentration of fluoxetine suspension was 0.13mg/ml, stored at low temperature at 4°C); (3) XYKAJY formulation group: XYKAJY was intragastric at the high dose of 1.95g/ml (XYKAJY-H) and low dose of 0.488 g/ml (XYKAJY-L) for 21 days. All of the groups had 12 mice alone.

### 2.4. Behavioral Tests

#### 2.4.1. Sucrose Preference

Sucrose preference test is a method of measuring anhedonia-like behavior. Mice had access to two 25 ml sipper tubes with different solutions, one containing 1% sucrose solution and the other containing normal drinking water. The location of the two sipper tubes should be switched daily. Before the start of experiment, mice housed individually were acclimated to the cages with sipper tubes for 3 days (days 1-3) with access to food and water, during which baseline measurements were taken. Sucrose preference was calculated as a percentage of the volume of 1% sucrose consumed over the total fluid intake volume. An individual cohort of mice (n=8 per group) was tested.

#### 2.4.2. Novelty Suppressed Feeding (NSF)

The NSF assesses stress-induced depressive behavior by measuring the incubation period of an animal to eat a familiar food in an aversive environment. Mice were deprived of food for 24 h before the test. After approximately 5 min for adaption, mice were allowed access to an unused preweighing food pellet in a clean test cage, containing fresh wood-chip bedding, which was placed directly in dim light. Each mouse was placed in a corner of the cage at the beginning of test, and a stopwatch was immediately started. The latency to eat (s) was recorded, as the mouse biting the pellet with the use of forepaws.

#### 2.4.3. Open Field Test

Open field procedure was designed to detect spontaneous locomotor activity [20] with minor modifications. A wooden box (50 cm × 50 cm × 25 cm) with smooth surface was used in the study. For each trial, animals facing the wall of box were placed into one of the corners, then they were permitted to explore the environment ad libitum. The total locomotor activity was recorded for 5 min.

#### 2.4.4. Forced Swimming (FS) Test

The FS test was carried out to observe the behavior changes of animals in the state of despair. The behavioral cylinder (40 cm high, 25 cm in diameter) was filled with 24–25°C water to the height of 30 cm. Before the test, mice were allowed to swim for adaption in 1 min. The total immobile time during the 5-min test was recorded. Mice were considered inactive when the limbs of mice are stationary [21]. Then, the mouse was removed from the cylinder and dried by towels.

#### 2.4.5. Tail Suspension Test

This test assesses learned helplessness by suspended immobility [22]. Mice were suspended by tail and adhered to a rigid plastic sheet mounted approximately 30 cm for 5 min. The immobile time was recorded.

### 2.5. Tissue Collection

After the behavioral test and following deep CO_2_ asphyxiation, cardiac puncture was used to sample blood through heparin-lined syringes for corticosterone (CORT), Corticotropin releasing hormone (CRH), Adrenocorticotropic hormone (ACTH), also for breast cancer markers.

Half part of hippocampus was immediately dissected out and frozen for later protein expression assessment. Half part of hippocampus was immediately dissected out and homogenized, supernatant after centrifugation, placed in -80°C, keeping it for later testing of monoamine neurotransmitter. Tumors were aseptically removed and weighed.

### 2.6. Plasma CORT, CRH, and ACTH Concentrations

The concentrations of CORT, CRH, and ACTH were detected to determine potential differences among treatment groups in this model. Blood was kept in 4°C and centrifuged (15 min at 2,000 rpm), then the plasma was stored at -80°C until assayed. CORT, CRH, and ACTH were measured according to the manufacturer's instructions by Elisa (Enzo Life Sciences, Plymouth Meeting, PA, USA) after 1:40 dilution.

### 2.7. The Content of Monoamine Neurotransmitters

Monoamine neurotransmitters are considered to be the classic indicators of depression. The hippocampus is homogenized by electric homogenizer then centrifuged (10 min at 3,000 rpm), and supernatant was stored at -80°C until assayed. NE, DA, and 5-HT were detected by Elisa method.

### 2.8. The Content of Tumor Markers CEA, CA125, and CA153

Carcinoembryonic antigen (CEA), carbohydrate antigen (CA125), and carbohydrate antigen (CA153) can be used as the basis for evaluating the prognosis of breast cancer. CEA, CA125, and CA153 were detected by Elisa method, specific process referred to instructions for the kit.

### 2.9. Histology

Brain tissues were decalcified in 4% polyoxymethylene at room temperature for 3-4 days and then dehydrated and embedded in paraffin. Hematoxylin and eosin (H&E) staining was used to quantify tumor, hippocampal in 5-*μ*m paraffin sections. Histopathological changes were detected by the Bioquant system imaging software (Bioquant Image Analysis Co., Nashville, TN, USA).

### 2.10. Western-Blotting

Radio immunoprecipitation assay (RIPA) and phenylmethylsulfonyl fluoride protease inhibitor (Solarbio Science and Technology, Beijing, PRC) were used to obtain protein. Protein concentrations were analyzed using nucleic acid protein detector (England, BioDrop). Proteins (50 *μ*g) were resolved by electrophoresis at a constant voltage of 120V for 100 minutes on sodium dodecyl sulfate (SDS)–polyacrylamide gels and transferred to polyvinylidene fluoride (PVDF) membrane (Millipore, MA). The membranes were incubated with different antibodies (1:1000 monoclonal rabbit anti-GR, anti-NR2A, anti-BDNF (Abcam), 1:1500 monoclonal rabbit anti-NR2B, anti-CaMKII, anti-CREB (Abcam), overnight at 4°C, and then with the appropriate secondary anti-body in the following day (1:2,000; Beijing Biosynthesis Biotechnology, Beijing, PRC) for 1 hour at room temperature. Immunoreactive bands were quantified by scanning densitometry (Quantity One software; Bio-Rad), and the density of each band was normalized to that of its own GAPDH.

### 2.11. Statistical Analysis

Data are presented as means ± SEM. One-way analysis of variance (ANOVA) and t-tests were used for comparisons among and between groups, respectively. For all analyses carried out using SPSS 16.0, P<0.05 was considered to indicate a statistically significant difference.

## 3. Results

### 3.1. XYKAJY Formulation Improved Depressive Symptoms

**Figure 1:**
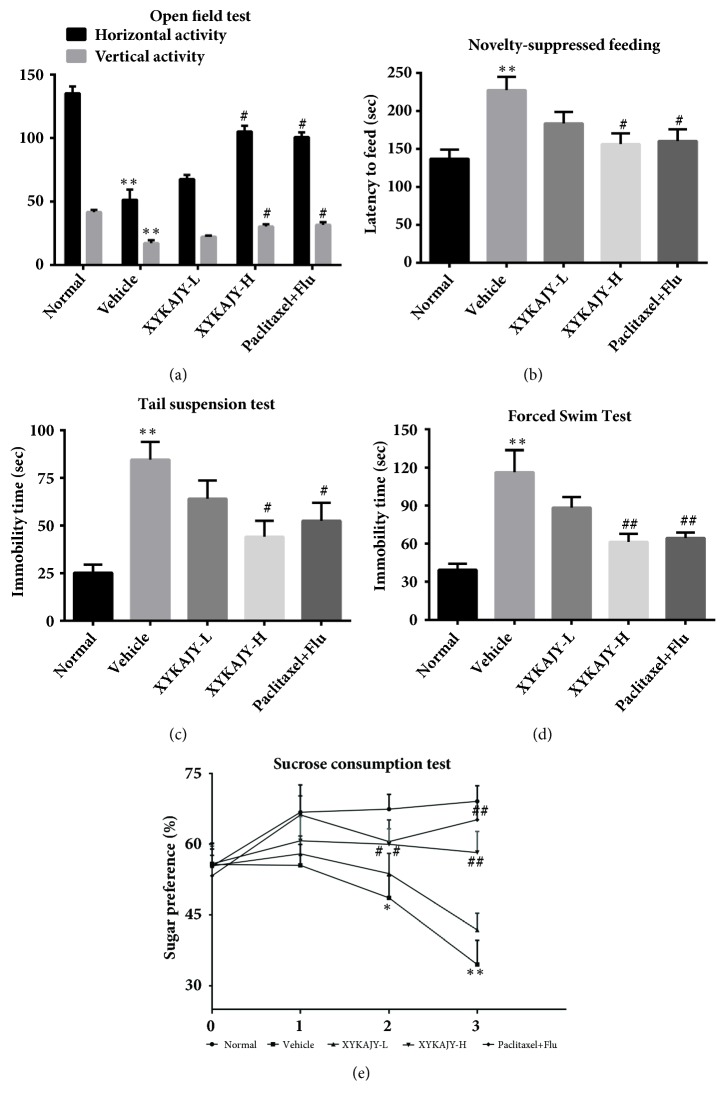
The behavior scores in normal group, vehicle group, XYKAJY-L group, XYKAJY-H group, and paclitaxel combined with fluoxetine group were expressed as mean ± S.E.M. (n = 10). (a) Horizontal and vertical activity of open field test; (b) latency to feed(s); (c) immobility time of tail suspension test; (d) immobility time of forced swim test; (e) sucrose preference (%). Compared with normal group: *∗P* < 0.05; *∗∗ P* < 0.01; compared with vehicle group: #* P* < 0.05; ##* P* < 0.01.

Figure 1(a) shows the change of autonomic activity. There was statistical decrease in the autonomic activity of vehicle group (*P* < 0.05). XYKAJY-H increased the times of autonomic activity (*P* < 0.01 or* P* < 0.05), as well as paclitaxel combined with fluoxetine. While XYKAJY-L had a certain influence on the autonomic activity of mice, but there was no statistical significance. It is suggested that XYKAJY formulation can significantly improve the depressive autonomic behavior.

Figure 1(b) shows the change of environmental inquiry. The feeding latency could reflect the desire to explore and curiosity about the unknown environment. The feeding latency was statistically increased in vehicle group (*P* < 0.05). There was significant decrease in the feeding latency in the group of XYKAJY-H and paclitaxel combined with fluoxetine (*P* < 0.05), while there is no statistically significant difference in XYKAJY-L group. This data demonstrated that the effects of exploration and feeding in novelty could be effectively relieved by treatment with XYKAJY.

Figures 1(c) and 1(d) show the change of desperate behavior. The total immovable time in the tail suspension test and forced swimming test increased in the vehicle group; the difference was statistically significant (*P* < 0.01), while XYKAJY-H and paclitaxel combined with fluoxetine decreased total immovable time significantly (*P* < 0.01 or* P* < 0.05). There is no statistical difference in the total immovable time of tail suspension and forced swimming test about XYKAJY-L group. The high dose of XYKAJY formulation revealed more significant therapeutic effectiveness as compared to positive control.

Figure 1(e) shows the effect of sugar preference, which represented the core symptoms of depression. The sugar water consumption of vehicle group in the first week was not statistically significant, while it significantly decreased in the second and third weeks (P < 0.01 or P < 0.05), which suggested the loss of sugar water consumption was decreased as the time goes on modeling. XYKAJY-H and paclitaxel combined with fluoxetine significantly increased 1% sugar water consumption in the second and third weeks (P < 0.05), also there was no statistical significance in the second week of XYKAJY-L group. It suggested that XYKAJY formulation could improve the responsiveness of cancer-related depression concurrent with breast cancer mice to pleasure.

### 3.2. XYKAJY Prescription Elevates the Content of Monoamine Neurotransmitters

**Figure 2:**
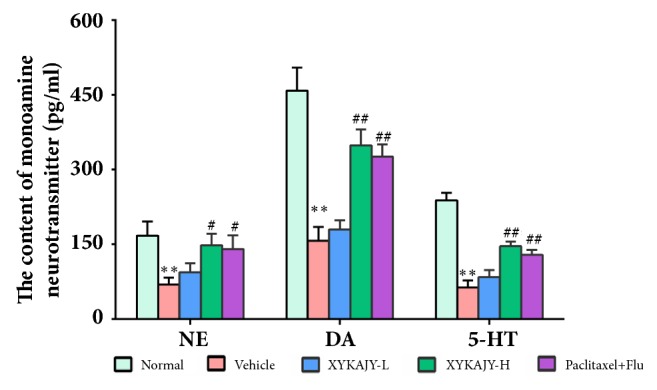
The content of NE, DA, and 5-HT in normal group, vehicle group, XYKAJY-L group, XYKAJY-H group, paclitaxel combined with fluoxetine group was expressed as mean ± S.E.M. (n = 10). Compared with normal group: *∗P* < 0.05; *∗∗ P* < 0.01; compared with vehicle group: #* P* < 0.05; ##* P* < 0.01.

The monoamine neurotransmitters NE, DA, and 5-HT decreased significantly in the vehicle group, and the difference was statistically significant (P < 0.01), while the monoamine neurotransmitters were obviously increased in the group of XYKAJY-H and paclitaxel with fluoxetine (P < 0.01 or P < 0.05). It was suggested that XYKAJY formulation can significantly increase the expression of monoamine neurotransmitter in hippocampus in order to achieve its antidepressant effect (Figure 2).

### 3.3. XYKAJY Formulation Restrain Breast Tumor Proliferation and Decrease the Content of Tumor Markers

**Figure 3:**
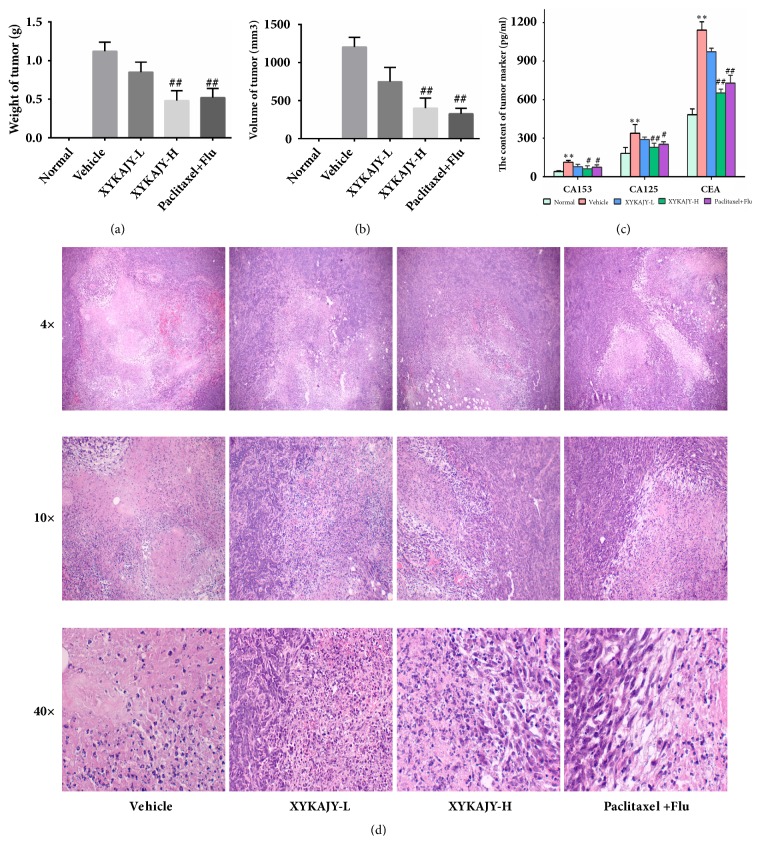
The proliferation of breast tumor and the content of tumor markers in normal group, vehicle group, XYKAJY-L group, XYKAJY-H group, and paclitaxel combined with fluoxetine group were expressed as mean ± S.E.M. (n = 10). (a) Weight of breast tumor; (b) volume of breast tumor; (c) content of CA153, CA125, and CEA; (d) structure of breast tumor under light microscope. Compared with normal group: *∗P* < 0.05; *∗∗ P* < 0.01; compared with vehicle group: #* P* < 0.05; ##* P* < 0.01.

The weight and volume of breast tumor decreased significantly in the group of XYKAJY-H and paclitaxel with fluoxetine (P < 0.01 or P<0.05), also the tumor suppressor rate was obviously increased (P < 0.01 or P<0.05), which was more than 50% in the group of XYKAJY and paclitaxel combined with fluoxetine, suggesting that XYKAJY inhibits the proliferation of breast tumor (Figures 3(a) and 3(b)).

The tumor markers CA153, CA125, and CEA in the vehicle group increased significantly (P < 0.01). While CA153, CA125, and CEA were significantly decreased after treatment with XYKAJY-H and paclitaxel combined with fluoxetine (P < 0.01 or P < 0.05), which suggested that XYKAJY can reduce the expression of tumor markers CA153, CA125, and CEA (Figure 3(c)). Also, there was no significant statistical difference of XYKAJY-L group.

Observing the structure of breast tumor under light microscope, different pathological changes were seen in the vehicle group; tumor cells arranged closely and the necrosis decreased, while the nuclear division increased, and also the blood vessels filled in tumor. After administration of XYKAJY and paclitaxel combined with fluoxetine, there were large areas of necrosis and cell debris, the gap became larger along the living cells, and the mitotic phenomenon decreased, suggesting that XYKAJY has the function of protecting the tumor cell and reducing proliferation (Figure 3(d)).

### 3.4. XYKAJY Formulation Depress Related Indexes of HPA Axis

**Figure 4:**
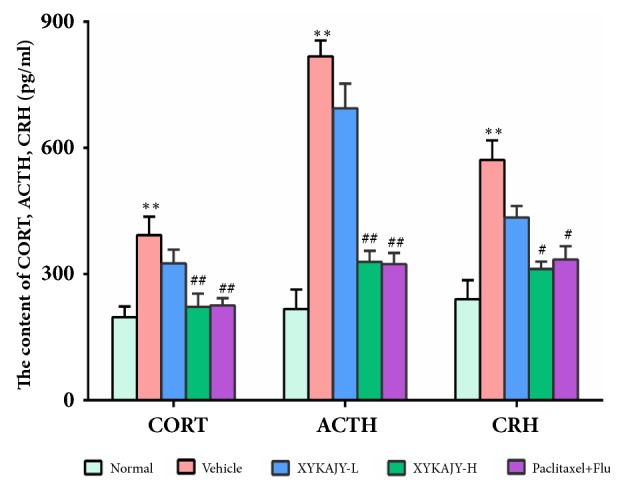
The content of CORT, ACTH, and CRH in normal group, vehicle group, XYKAJY-L group, XYKAJY-H group, and paclitaxel combined with fluoxetine group was expressed as mean ± S.E.M. (n = 10). Compared with normal group: *∗P* < 0.05; *∗∗ P* < 0.01; compared with vehicle group: #* P* < 0.05; ##* P* < 0.01.

The levels of CORT, ACTH, and CRH in peripheral blood directly correlate with the HPA axis reactivity and reflect the state of the body under stress [23, 24]. CORT, ACTH, and CRH were significantly elevated in vehicle group (*P* < 0.01 or* P* < 0.05). However, they were significantly decreased after being treated with XYKAJY-H and paclitaxel combined with fluoxetine (*P* < 0.01). These results suggest that the expression of CORT, ACTH, and CRH in the serum of vehicle mice and the hyperfunction of HPA axis was reversed by XYKAJY (Figure 4).

### 3.5. XYKAJY Formulation Protect Pathological Structure of Hippocampal

**Figure 5:**
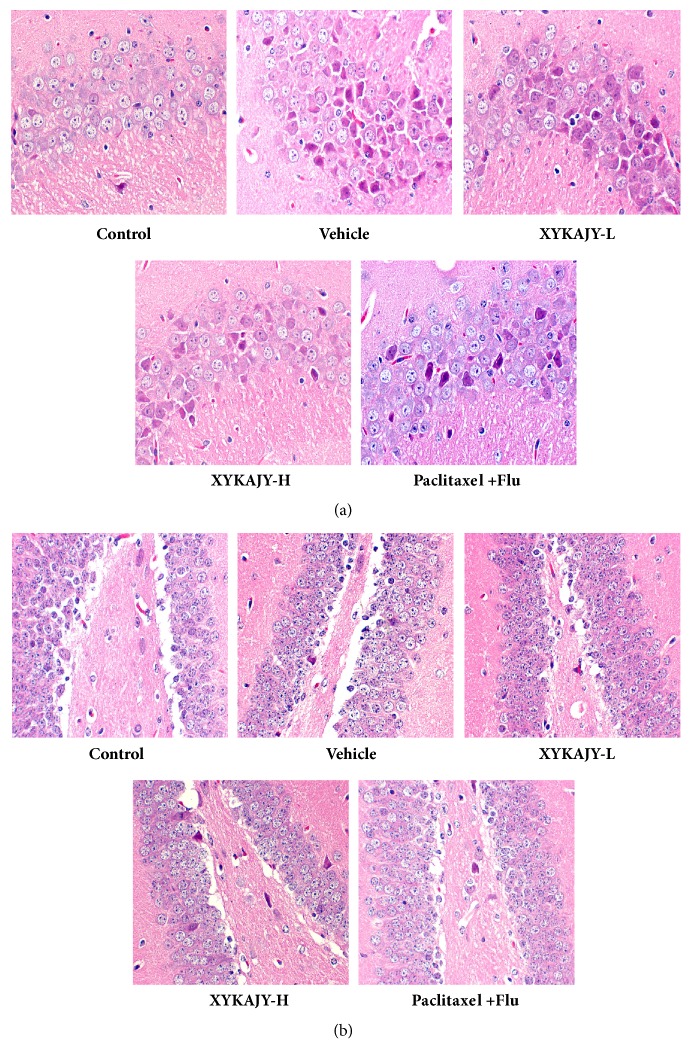
The pathological structure of hippocampal in normal group, vehicle group, XYKAJY-L group, XYKAJY-H group, and paclitaxel combined with fluoxetine group. (a) CA3 area of hippocampal; (b) DG area of hippocampal.

Hippocampus is the center of regulating the function of HPA axis. The neurons in the vehicle group showed atrophy, cytoplasm concentration, nuclear deep dye, and cell necrosis as compared to normal control. XYKAJY-H, paclitaxel combined with fluoxetine groups showed clear cell boundaries, structural and nucleus morphology, while cell nuclear staining and cell shrinkage were seen in the XYKAJY-L group, which reflected better characteristics of the protection of the hippocampus about XYKAJY-H. It suggested that the CA3, DG of hippocampal in vehicle mice is damaged, while XYKAJY relieves the damage of neurons in the hippocampus (Figure 5).

### 3.6. XYKAJY Formulation Regulate the Expression of GR, NR2A, NR2B, CAMKII, BDNF, and CERB in Hippocampal

GR is important index for evaluating the function of the HPA axis. Meanwhile NR is a connection point between HPA axis and hippocampus, having two subtypes NR2A and NR2B, which are directly related to protein on regulating synaptic plasticity. Synaptic plasticity can be regulated by neurotrophic and calmodulin, such as BDNF, CERB, and CAMKII, also they are downstream proteins followed by NR.

**Figure 6:**
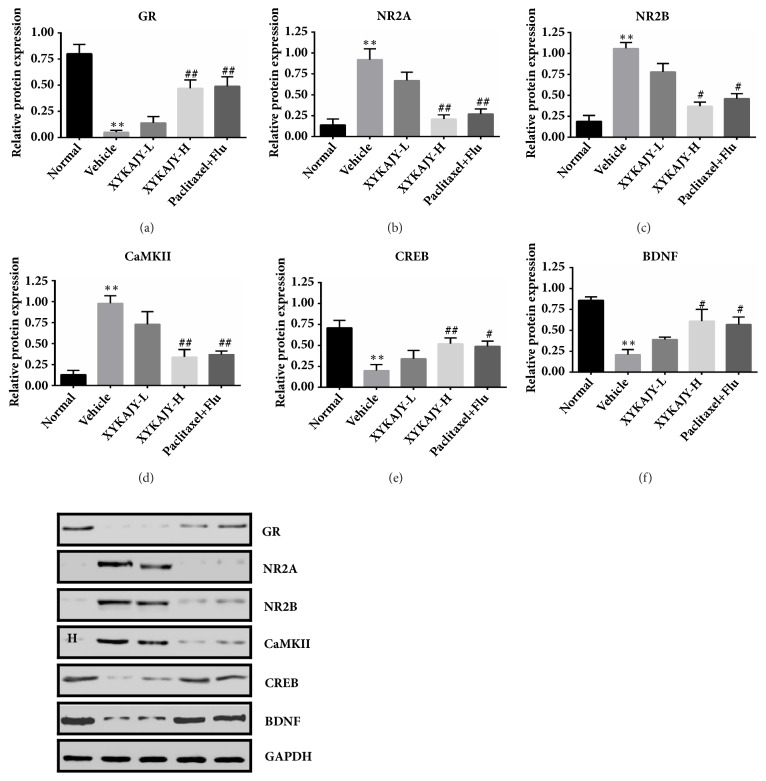
The protein expression was examined by western-blotting. The expression of GR, NR2A, NR2B, CAMKII, BDNF, and CERB in normal group, vehicle group, XYKAJY-L group, XYKAJY-H group, and paclitaxel combined with fluoxetine group was expressed as mean ± S.E.M. (n = 6). Quantification of bands was quantified using the image J program with GAPDH as a normalized control. Compared with normal group: *∗P* < 0.05; *∗∗ P* < 0.01; compared with vehicle group: #* P* < 0.05; ##* P* < 0.01.

In our study, the expression of GR, BDNF, and CREB decreased in the vehicle group (*P* < 0.01), while the expression of NR2A, NR2B, and CaMKII increased (*P* < 0.01). XYKAJY-H, paclitaxel combined with fluoxetine significantly increased the expression of GR, BDNF, and CREB (*P* < 0.01,* P* < 0.05) and decreased the expression of NR2A, NR2B, and CaMKII in hippocampal (*P* < 0.01,* P* < 0.05) (Figure 6).

## 4. Discussion

Breast cancer is one of the most common cancers worldwide, which is influenced by various factors. Patients are sensitive to mental health problems with nearly 50% suffering depression or anxiety [25]. According to the research report, 10–20% breast cancer patients experienced a major depressive episode after diagnosis [26].

The detection of serum tumor markers has important significance for the occurrence, development, treatment, and prognosis of breast cancer. Serum CEA, CA153, and CA125 are a kind of tumor markers widely used in clinical diagnosis of breast cancer. In our study, XYKAJY formulation reduces the content of CEA, CA153, CA125, weight, and volume of tumor. The results indicate that Traditional Chinese medicine XYKAJY formulation has effect of inhibiting the proliferation of breast cancer to some extent. Interestingly, we found breast tumor produces obvious necrosis-like symptoms, when hypodermic injection of corticosterone after 4T1 cells subaxillary injected and formatted an obvious tumor. Chemotherapy combined with fluoxetine did not reduce or inhibit the appearance of necrotic tissue, while XYKAJY formulation significantly reduces the production of necrotic like tissue obviously. Its mechanism is unclear, and how the corticosterone causes necrosis-like tissue formation will be the focus of our follow-up research.

In this study, XYKAJY formulation effectively improves the sugar preference and autonomic activity, reduces the incubation period of feeding, and also reverses the typical depressive mental state of vehicle mice. Monoamine neurotransmitters are considered to be the markers of depression, and their changes can explain the severity of depression [27]. Simultaneously, the content of monoamine neurotransmitters, such as 5-HT, NE, and DA, was increased significantly after the treatment of XYKAJY formulation. As a result, our experiment shows XYKAJY formulation plays a good antidepressant effect in cancer-related depression concurrent with breast cancer.

Cancer patients suffer from diverse degree of affective and cognitive disturbances, which have been attributed to the combination of tumor biology, cancer therapy, and stress [28]. However, these different factors are difficult to be distinguished clinically, and also their possible mechanism of interaction is still entangled or ignored. Enduring immune disorders and inflammatory activation correlated with persistent negative emotions in breast cancer [29], suggesting a potential immune mechanism about cancer-related depression. Furthermore, glucocorticoids and their responses to multiple challenges are interrupted among cancer survivors [30]. Because glucocorticoids have an effective anti-inflammatory activity, they are also assumed to be associated with persistent behavioral complications [31].

Tumors may cause long-term changes in immune, HPA axis, or nervous systems, which lead to the continued depressed behavioral symptoms of breast cancer patients, yet neurobiological mechanism in this population remains largely unexamined. Atypical functioning of HPA axis is generally believed to be closely related with depression, which is a component of the body's physiological stress response system. The hyperactivity of HPA axis also exists in breast cancer patients. Reduction in depressive-like symptoms and a normalization of HPA axis regulation have great significance for long-term survival in women recovering from early-breast cancer treatment [32]. Taken together, the function of HPA axis is as an indicator of both psychological distress and circadian rhythm disruption which have repeatedly been linked with morbidity and mortality in cancer [33]. Our findings suggested that XYKAJY formulation exerts antidepressant effect, and its potential mechanism may be implemented through reversing the high degree of HPA axis.

The imbalance of HPA axis causing the damage of hippocampal neurons is a major driving force of depression, as hippocampus is a key region of regulating emotion, learning, and memory [34]. Glucocorticoids affect hippocampal structure, acquisition of memory, and regulation of emotion. At the same time, glucocorticoids receptors are most abundant in the hippocampus, which can negatively regulate the function and activity of HPA axis [35]. The disturbance of HPA axis causes increasing of COR and CRH continuously and also decreasing of the expression of glucocorticoids receptor, which leads to the accumulation of intracellular glutamic acid (Glu) and release of the extracellular further, then activating NR2A, NR2B, which are the subunits of NR binding protein, causing neuronal excitotoxic further in the hippocampus [36].

NR is a glutamate sensitive ion channel receptor distributed in hippocampal, which is an important regulatory protein of hippocampal synaptic plasticity [37]. Abnormal activation of NR promotes the increase of Ca2+ influx in cells, which causes the imbalance of Ca2+ homeostasis [38], then affects the synaptic plasticity, dendritic morphology, and also the pathological process of neuronal death [39]. In addition, a large number of Ca2+ inflows phosphorylate CaMKII [40] and inhibit the transcriptional activity of CREB further [41], which reduce neurotrophic in hippocampal, such as brain derived neurotrophic factor (BDNF) and neural growth factor (NGF), causing hippocampal neurons to damage thereby [42]. Our data showed that after the treatment of XYKAJY formulation, the pathological structure of DG, CA3, and adrenal tissues trends to normal degree, and the expression of NR2A, NR2B, CaMKII was significantly downregulated, while the expression of CREB, GR, BDNF was significantly upregulated. These data demonstrated that XYKAJY exerts its antidepressant effect by promoting hippocampal synaptic plasticity.

Currently, there is no specific drug for treating cancer-related depression concurrent with breast cancer, also the combination of chemical synthetic drugs has different side effects. Chinese herbal compound is expert in overall adjustment and has unique characteristics for the treatment of complications. XYKAJY formulation is derived from Xiaoyao San, which has a long history of antidepressant effect in China and is listed in the Chinese authoritative “Pharmacopoeia”. Our study shows that XYKAJY formulation inhibits the proliferation of breast tumor to some extent, also effectively alleviates the depressive behavior, and protects the structure and function of the hippocampus, and its potential mechanism may be through promoting hippocampal synaptic plasticity.

## 5. Conclusion

Our study demonstrated that XYKAJY formulation improved the autonomic behavior, reduced the incubation period of feeding, and reversed the typical depressive-like symptoms in breast cancer mice. Furthermore, XYKAJY formulation was found to reduce hyperfunction of HPA axis and the content of breast cancer marker in the blood, promote the content of monoamine neurotransmitter, and protect the pathological changes of the hippocampus and tumor, as well as upregulating the expression of GR, CREB, and BDNF in the hippocampus and decreasing NR2A, NR2B, CaMKII. These results provide experimental evidence supporting that XYKAJY formulation effectively alleviates depression-like behaviors and tumor proliferation by protecting the structure and function of the hippocampus synaptic plasticity.

## Figures and Tables

**Table 1:** Components of XYKAJY formulation.

Chinese name	Botanical name	Amount (g)
Chai Hu	*Bupleurum chinense DC.*	9
Dang Gui	*Angelica sinensis (Oliv.) Diels*	9
Bai Shao	*Paeonia lactiflora Pall.*	9
Bai Zhu	*Atractylodes macrocephala Koidz.*	9
Xia Ku Cao	* Prunella vulgaris L.*	15
Chong Lou	*Paris polyphylla Smith var.*	9
Ren Shen	* Panax ginseng C.A. Mey.*	6
Jiang Huang	*Curcuma Longa L.*	6
Guan Ye Lian Qiao	*Hypericum perforatum L.*	6
Pu Gong Ying	*Taraxacum mongolicum Hand.-Mazz.*	6
Bai Hua She She Cao	* Hedyotis diffusa Willd.*	6
Zhi Gan Cao	*Glycyrrhiza uralensis Fisch.*	6

## Data Availability

The data used to support the findings of this study are available from the corresponding author upon request.
